# Intravenous Immunoglobulin Therapy Eliminates *Candida* *albicans* and Maintains Intestinal Homeostasis in a Murine Model of Dextran Sulfate Sodium-Induced Colitis

**DOI:** 10.3390/ijms20061473

**Published:** 2019-03-23

**Authors:** Rogatien Charlet, Boualem Sendid, Srini V. Kaveri, Daniel Poulain, Jagadeesh Bayry, Samir Jawhara

**Affiliations:** 1 Inserm, U995/Team2, Université Lille, 1 place Verdun, F-59000 Lille, France; charlet-rogatien@hotmail.fr (R.C.); boualem.sendid@univ-lille.fr (B.S.); daniel.poulain@univ-lille.fr (D.P.); 2 University Lille2, U995-LIRIC, Lille Inflammation Research International Centre, F-59000 Lille, France; 3 CHU Lille, Service de Parasitologie Mycologie, Pôle de Biologie Pathologie Génétique, F-59000 Lille, France; 4 Inserm Centre de Recherche des Cordeliers, Equipe-Immunopathologie et Immuno-intervention Thérapeutique, Sorbonne Universités, Université Paris Descartes, Sorbonne Paris Cité, F-75006 Paris, France; srini.kaveri@crc.jussieu.fr

**Keywords:** intravenous immunoglobulin G, colitis, dextran sulfate sodium, mice, inflammation, cytokines, *Candida albicans*, *Escherichia coli*, *Enterococcus faecalis*

## Abstract

Intravenous immunoglobulin (IVIg) therapy has diverse anti-inflammatory and immunomodulatory effects and has been employed successfully in autoimmune and inflammatory diseases. The role of IVIg therapy in the modulation of intestinal inflammation and fungal elimination has not been yet investigated. We studied IVIg therapy in a murine model of dextran sulfate sodium (DSS)-induced colitis. Mice received a single oral inoculum of *Candida* *albicans* and were exposed to DSS treatment for 2 weeks to induce colitis. All mice received daily IVIg therapy starting on day 1 for 7 days. IVIg therapy not only prevented a loss of body weight caused by the development of colitis but also reduced the severity of intestinal inflammation, as determined by clinical and histological scores. IVIg treatment significantly reduced the *Escherichia* *coli,* *Enterococcus* *faecalis*, and *C.* *albicans* populations in mice. The beneficial effects of IVIg were associated with the suppression of inflammatory cytokine interleukin (IL)-6 and enhancement of IL-10 in the gut. IVIg therapy also led to an increased expression of peroxisome proliferator-activated receptor gamma (PPARγ), while toll-like receptor 4 (TLR-4) expression was reduced. IVIg treatment reduces intestinal inflammation in mice and eliminates *C.* *albicans* overgrowth from the gut in association with down-regulation of pro-inflammatory mediators combined with up-regulation of anti-inflammatory cytokines.

## 1. Introduction

*Candida albicans* infections continue to be a serious clinical problem in terms of their high morbidity and mortality [1,2]. The interaction between the fungus and its host occurs at the level of the cell wall, which consists mainly of polysaccharides associated with proteins and lipids. Its innermost layers are composed of a dense network of polysaccharides consisting of glucans (β-1,3 and β-1,6 linked glucose) and chitin (a polymer of β-1,4-linked N-acetylglucosamine) [3]. Fungal polysaccharides are shed into the circulation during infection, and their detection enables the early diagnosis of invasive fungal infection [1]. Clinical and experimental studies have shown that *C. albicans* infections can generate anti-glycan antibodies known as ASCA (anti-*Saccharomyces cerevisiae* mannan antibodies) [4]. These anti-fungal glycan antibodies were initially described as serological markers of Crohn’s disease (CD), but subsequent studies have established that they can also be generated during *Candida* infection, suggesting a link between CD gut dysbiosis and endogenous opportunistic fungal species [5,6]. CD and ulcerative colitis (UC) are the main forms of inflammatory bowel disease (IBD). CD and UC are distinguishable by the location of the inflammation and by the pattern of histological alterations in the gastrointestinal (GI) tract. Animal models have played a significant role in increasing our understanding of IBD pathogenesis, especially models of murine colitis [7]. Experimental studies have shown that *C. albicans* exacerbates the intestinal inflammation induced by dextran sulfate sodium (DSS) in mice, and, conversely, that DSS colitis promotes fungal colonization [8,9].

Immunotherapy with intravenous immunoglobulin (IVIg) is widely used in the management of various autoimmune and inflammatory diseases [10,11,12]. Experimental studies and evaluation of patients undergoing IVIg therapy show that the beneficial effects of IVIg in inflammatory and autoimmune diseases involve diverse mechanisms, including inhibition of activation of innate immune cells and release of inflammatory cytokines, reciprocal regulation of effector T-cells (Th1 and Th17) and regulatory T-cells (Tregs), neutralization of autoantibodies and complement, and suppression of autoantibody production [13,14].

Several case studies and open-label trials showed that IVIg could induce significant improvement in aminosalicylate- and steroid-resistant CD [15]. Although the number of patients is small, the data imply that IVIg could induce swift relief from inflammation and significantly improve these patients without causing side-effects. However, the mechanism by which IVIg therapy benefits CD patients is currently unknown.

We investigated the protective effects of IVIg therapy in a murine model of DSS-induced colitis. By using clinical and histological parameters, we reported that IVIg therapy reduced the intestinal inflammation and was associated with the prevention of a loss of body weight. Further, IVIg therapy significantly reduced the burden of *C. albicans* in the intestine and other organs. The beneficial effects of IVIg in DSS-induced colitis were associated with the suppression of inflammatory cytokine interleukin (IL)-6 and enhancement of IL-10 in the gut. IVIg therapy also led to an increased expression of peroxisome proliferator-activated receptor gamma (PPARγ), while toll-like receptor 4 (TLR-4) expression was reduced. These data provide experimental evidence for the therapeutic utility of IVIg in IBD.

## 2. Results

### 2.1. IVIg Treatment Reduces the Severity of Intestinal Inflammation in C. albicans Colonized Mice with DSS-Induced Colitis

**Figure 1:**
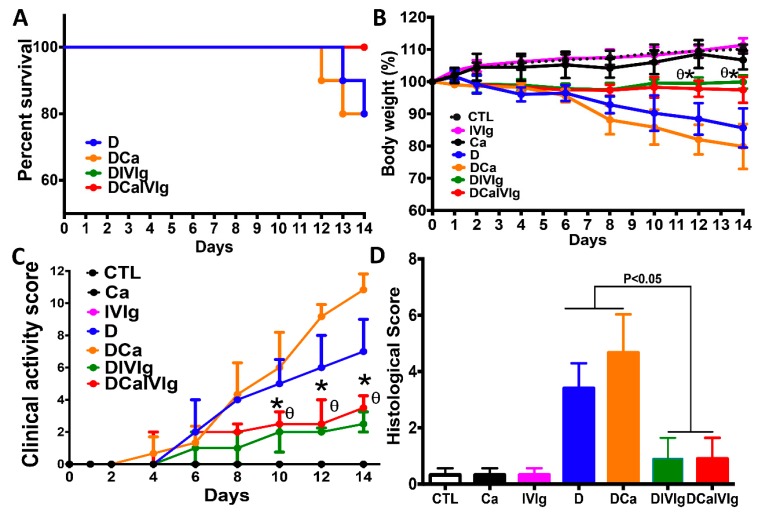
Effect of intravenous immunoglobulin (IVIg) treatment on the survival of mice, body weight, and inflammatory scores in the dextran sulfate sodium (DSS)-induced colitis model. (**A**) Mouse survival. Results are expressed as percent survival from the time of *C. albicans* challenge and DSS treatment. CTL, Ca, IVIg or D correspond to control groups receiving water, *C. albicans*, IVIg treatment or DSS, respectively. DCa corresponds to mice receiving *C. albicans* and treated with DSS. DIVIg corresponds to mice receiving DSS and treated with IVIg. DCaIVIg represents mice receiving *C. albicans* challenged with IVIg and treated with DSS. No mouse mortality was recorded in the Ca or IVIg groups, while DSS treatment induced 20% mouse mortality in groups D and DCa; (**B**) Body weight. Results are expressed as a percent; (**C**) Clinical analysis of DSS-induced colitis in mice after IVIg treatment. Clinical score was determined by assessing weight loss, change in stool consistency, and presence of gross bleeding. The clinical score ranged from 0 to 12 (each value corresponds to the mean value over 14 days per group). * *p* < 0.05 for DCaIVIg mice vs. DCa and D mice; (**D**) Histological scores. Scores range from 0 (no changes) to six (extensive cell infiltration and tissue damage). Data are the mean ± SD of 14 mice per group.

In order to analyze the efficiency of IVIg in the modulation of intestinal inflammation, mice received 2% DSS for 2 weeks in drinking water to promote the development of colitis. IVIg (Sandoglobulin) was administered via intraperitoneal injection (0.8 g/kg/day for 7 days) to mice treated with DSS and challenged with *C. albicans*. Mice treated with DSS or DSS + *C. albicans* had a mortality rate of approximately 20%, while no mortality was recorded in mice treated with IVIg alone (Figure 1A).

DSS treatment led to a 15% loss of body weight of mice at 2 weeks (Figure 1B). Although *C. albicans* did not alter the body weight of mice in the absence of DSS-induced colonic inflammation, *C. albicans* triggered a further loss of body weight (nearly 20%) in DSS-induced colitis, while mice treated with DSS solely displayed 15% of body weight loss. In both groups of mice, the clinical scores increased significantly from day 7 onwards (Figure 1C). However, IVIg therapy not only rescued the mice from body weight loss but also significantly reduced the severity of intestinal inflammation, as determined by clinical and histological scores in DIVIg or DCaIVIg mice.

**Figure 2:**
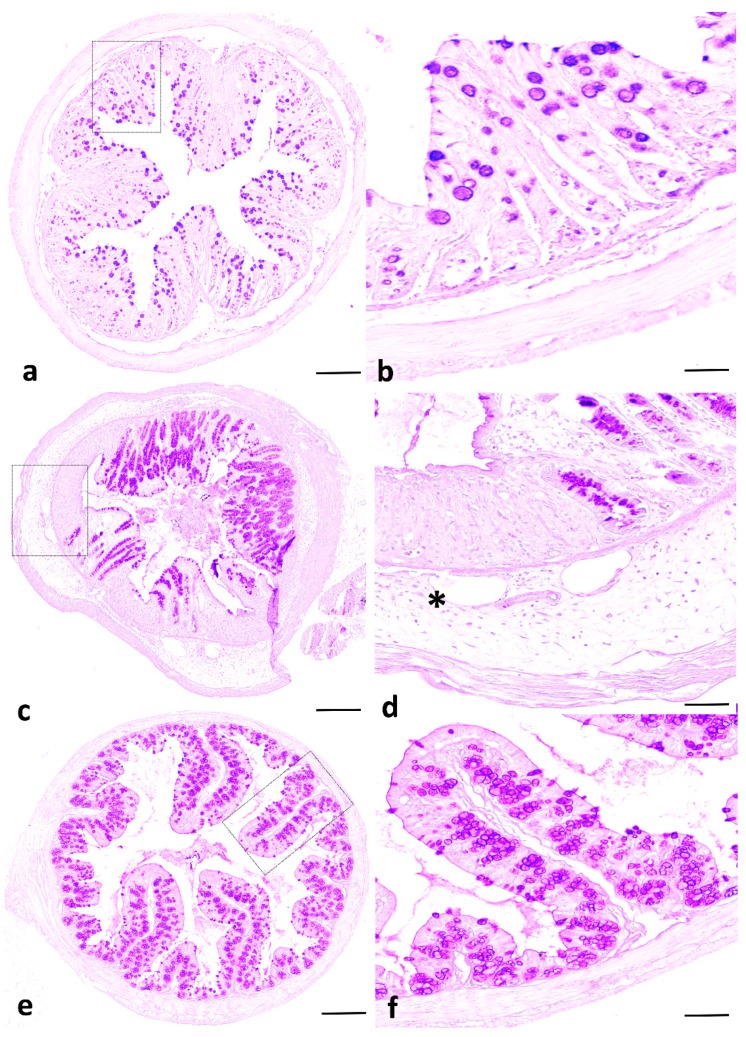
Histological analysis of colons from mice with dextran sulfate sodium (DSS)-induced colitis. Panels (**a**,**b**) display sections from mice that received *C. albicans;* panels (**c**,**d**) correspond to *C. albicans* + DSS; and panels (**e**,**f**) denote colon sections from mice that received *C. albicans* + DSS + intravenous immunoglobulin (IVIg). *C. albicans* alone did not induce any epithelial injury or an inflammatory cell infiltrate in the colon wall structure. Colon sections from DCa mice show an inflammatory cell infiltrate in colon wall structures and massive tissue destruction (asterisk), while colon sections from DCaIVIg mice show minimal tissue damage. The scale bars represent 50 µm (**a**,**c**,**e**) and 10 µm (**b**,**d**,**f**).

Histological analysis of the colon revealed that *C. albicans* alone did not induce either epithelial injury or inflammatory cell infiltration in the colon wall (Figure 1D). In DSS-induced colitis, *C. albicans* enhanced inflammatory cell infiltration in the colon wall structures and massive tissue destruction. However, IVIg protected the mice from this severe tissue destruction (Figure 2).

### 2.2. IVIg Treatment Decreases the Escherichia coli, Enterococcus faecalis, and C. albicans Populations in Mice with DSS-Induced Colitis

**Figure 3:**
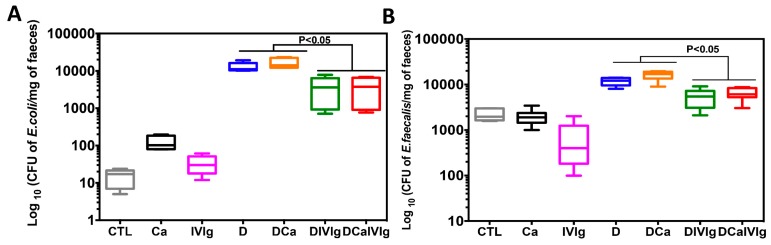
Determination of viable fecal aerobic bacteria in mice with colitis and treated with intravenous immunoglobulin (IVIg) (**A**,**B**) The number of *E. coli* and *E. faecalis* colonies recovered from colonic luminal contents. Data are expressed as box-and-whiskers plots, with min to the max range as whiskers. CFU: colony forming units.

**Figure 4:**
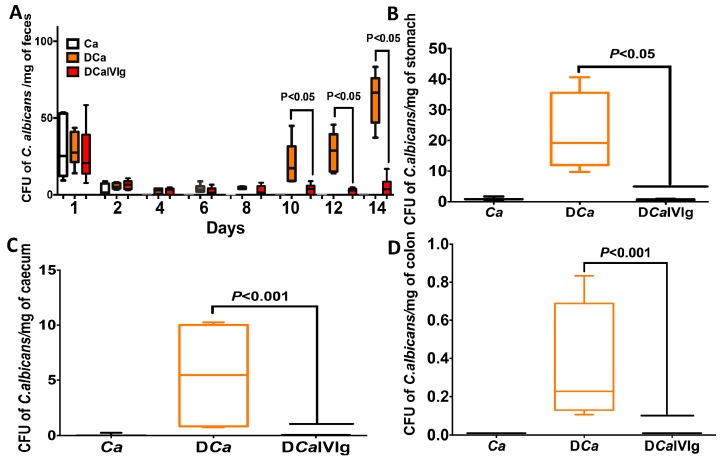
Evaluation of *C. albicans* colonization in various organs following intravenous immunoglobulin (IVIg) treatment of mice with dextran sulfate sodium (DSS)-induced colitis. (**A**) The number of *C. albicans* colony forming units (CFU) recovered from stools. Data are expressed as box-and-whiskers plots, with min to the max range as whiskers. (**B**–**D**) The number of *C. albicans* CFU recovered from the stomach, cecum, and colon. Data are expressed as box-and-whiskers plots, with min to the max range as whiskers.

To determine the effect of IVIg treatment on the growth of the *E. coli* and *E. faecalis* populations, the colonic luminal contents were analyzed at day 14 in all groups (Figure 3). We found that *C. albicans* alone or IVIg treatment, in the absence of colitis, did not induce any significant changes in the *E. coli* and *E. faecalis* populations, while DSS-induced colitis and *C. albicans* overgrowth promoted an increased *E. coli* and *E. faecalis* populations in mice. In contrast, IVIg treatment significantly reduced *E. coli* and *E. faecalis* in mice treated with DSS solely or DSS + *C. albicans* (Figure 3). The effect of IVIg treatment on *C.* albicans colonization was assessed in stool samples, stomach, and large intestine of mice (Figure 4). DSS promoted C. albicans colonization in the stomach, caecum, and colon. Thus, colonic inflammation induced by DSS promoted the establishment of *C. albicans* colonization. The number of *C. albicans* colony forming units (CFU) gradually increased during colitis development. Interestingly, IVIg treatment significantly reduced the burden of *C.* albicans and CFU of *C.* albicans in various organs (Figure 4).

### 2.3. IVIg Treatment Reduces Inflammatory Cytokines and Enhances IL-10 in the Colon

**Figure 5:**
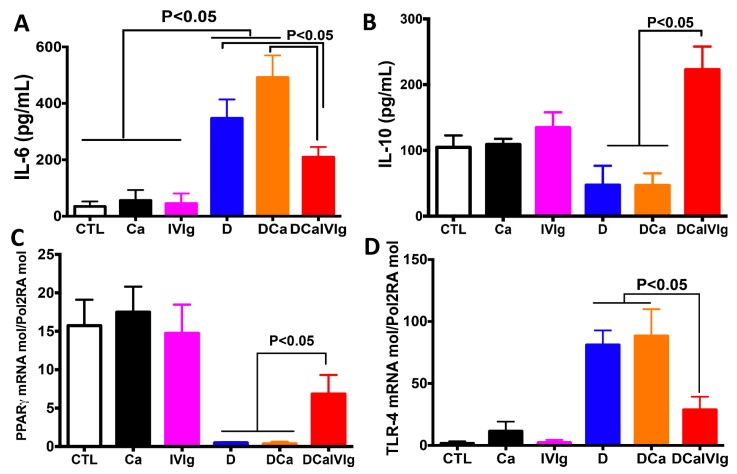
Cytokine and receptor expression after intravenous immunoglobulin (IVIg) treatment of mice with *C. albicans* and dextran sulfate sodium (DSS)-induced colitis. (**A**,**B**) Protein levels of interleukin (IL)-6 and IL-10 in mouse colons. (**C**,**D**) Relative expression of peroxisome proliferator-activated receptor gamma (PPARγ) and toll-like receptor 4 (TLR-4) mRNA in mouse colons. Data are the mean ± SD of 14 mice per group.

A significant reduction in the severity of intestinal inflammation, as measured by clinical and histological scores, suggests that IVIg treatment altered the inflammatory cytokines in the colon (Figure 5). IVIg significantly reduced IL-6 in the colon and was associated with a reciprocal enhancement of the anti-inflammatory cytokine IL-10. In addition, the transcript levels of PPARγ, a ligand-activated transcription factor that tilts the balance towards the production of anti-inflammatory mediators in innate cells, was significantly increased by IVIg. Interestingly, IVIg also reduced the transcript levels of TLR-4, in line with its role in intestinal inflammation (Figure 5).

## 3. Discussion

IVIg has diverse anti-inflammatory and immunomodulatory effects and has been employed successfully in autoimmune and inflammatory diseases. It is considered non-immunosuppressive and safe. By using the murine model of dextran sulfate sodium-induced colitis, we demonstrate that IVIg therapy not only reduces the intestinal inflammation but also eradicates *C. albicans* overgrowth from the gut. Although we used a mouse model, for the reasons discussed below, we employed human therapeutic normal immunoglobulin preparation rather than murine immunoglobulin for the therapy. IVIg is obtained from the pooled plasma of several thousand healthy donors (typically 3000 to 10,000 donors) with highly variable genetic, epigenetic, and environmental backgrounds. Reproducing these factors and the entire array of human immunoglobulin repertoire is quite impossible in mouse immunoglobulin preparation, and hence mouse immunoglobulin preparation equivalent of human IVIg is not available. As IVIg is infused at 1–2 g/kg body weight, obtaining such big quantities of mouse immunoglobulin is also a daunting task. On the other hand, the use of human IVIg has direct translational value for exploring this therapy in patients.

IVIg has been used in the treatment of CD and has been shown to bring about a rapid and clinically significant improvement in a patient’s condition. However, the role of IVIg in the modulation of intestinal inflammation and fungal elimination has not been yet studied. In the present study, low doses of DSS (2%) were administered to mice for 2 weeks to induce colonic inflammation and to promote the establishment of *C. albicans* overgrowth. In a pilot experiment, IVIg was administered to mice intraperitoneally, intravenously or orally. Intraperitoneal injection of IVIg after *C. albicans* challenge was more efficient at reducing inflammatory clinical signs than the other routes of administration (data not shown). This finding is consistent with previous reports showing that intraperitoneal injection is better than other routes in the setting of colitis amelioration [16,17].

IVIg has been used as an anti-infectious agent against viruses or bacteria in both patients and experimental models [18,19,20,21]. As IVIg is a pool of IgG from thousands of healthy donors, the exposure of individual donors to vaccinations, endemic infectious diseases, and ubiquitous microorganisms contribute to IgG antibodies against diverse microbes and their products [19,22,23]. These antibodies play an important role in the prevention of infectious episodes in primary immunodeficient patients. However, the beneficial effects of IVIg in infectious diseases go beyond simple neutralization of microbes or their toxins. Anti-inflammatory pathways are also critical for protection against infection [24]. IL-10 was shown to be critical for the protection rendered by IVIg against fatal herpes simplex virus (HSV) encephalitis in mice [25].

Investigations on IVIg therapy in fungal infections is, however, limited despite the fact that several compelling pieces of evidence have shown the protective role of immunoglobulins in the infections and inflammation mediated by several fungal species, including Candida, Aspergillus, Cryptococci, and others [26,27]. Antibodies either in the circulation of an individual that are formed due to natural exposure to fungi species or when used in the form of specific protective monoclonal antibodies could mediate protection against fungal infections by several mutually nonexclusive mechanisms, such as neutralization of fungi species and their pathogen-associated molecular patterns, alteration of expression of fungal transcripts, metabolism and signaling pathways, and suppression of formation of biofilm and liberation of polysaccharides. In addition, antibodies are also shown to enhance opsonization of fungi and promote complement activation and phagocytosis of fungi by innate cells like macrophages and monocytes [26].

Several observational studies have reported that infusion of IVIg reduces the incidence of fungal infections in immunocompromised patients. As IVIg is a pooled IgG purified from the plasma of several thousand healthy donors, these observations highlight the importance of anti-fungal IgG in the circulation to mediate resistance against fungal infections. Thus, prophylaxis IVIg therapy in hepatic allograft individuals receiving oral acyclovir led to a significant decrease in the occurrence of fungal infections [28]. Epidemiologic investigation in soldiers who received IVIg as a prophylactic vaccination reduced the rate of skin fungal infections [29]**.** Although not used in the form of IVIg, prophylaxis oral administration of bovine anti-Candida antibodies, that are obtained from the bovines immunized with many Candida species, were reported to reduce fungal colonization in the majority of the bone marrow transplant patients [30]. In line with these observations, our recent report demonstrates that IVIg protects from experimental allergic bronchopulmonary aspergillosis in mice [31]. Whether used as prophylaxis or therapy, IVIg in this model significantly reduced the *Aspergillus fumigatus* burden in the lungs. Of interest, this report also provided convincing evidence that beneficial effects of IVIg in fungal infections go beyond neutralization of fungi wherein we found that protective effects of IVIg were associated with reduced Th17 responses and concomitant enhancement of regulatory T cells and IL-10, the mechanisms implicated in the beneficial effects of IVIg in autoimmune conditions [32,33].

In the present study, IVIg promoted the elimination of *C. albicans* from the gut and reduced intestinal inflammation. This finding is also supported by decreased clinical and histological scores of inflammation. Although IVIg is known to contain IgG against *Candida* [34] and may help in the neutralization of *C. albicans*, the anti-inflammatory effects of IVIg appear to be crucial in the DSS-induced colitis model. In terms of the gut bacteria, we focused on *E. coli* and *E. faecalis* populations that are known to be involved in IBD [35,36]. *E. coli* and *E. faecalis* populations increased in mice that developed colitis, while the IVIg treatment reduced these aerobic bacteria. These observations are consistent with clinical and experimental studies, which show an increase in *E. coli* and *E. faecalis* in CD patients [36,37]. The beneficial effects of IVIg were associated with the suppression of inflammatory cytokine IL-6 and enhancement of IL-10 in the gut. Our data, along with previous data from an HSV encephalitis model, suggest that IL-10 plays a central role in mediating protection against the inflammation associated with infection.

In addition to cytokines, IVIg therapy also led to increased expression of PPARγ, a ligand-activated transcription factor that mediates anti-inflammatory functions and resolution of inflammation [38]. Previous studies have shown that IVIg suppresses the activation of monocytes/macrophages and neutrophils [39,40,41,42,43]. Clinically, blocking the pro-inflammatory cytokine stimulates IL-10 production by regulatory macrophages, which are involved in mucosal healing in IBD patients [44]. As PPARγ influences innate immune signaling and the induction of pro-inflammatory cytokines, including IL-6, it is plausible that increased expression of PPARγ might contribute to the anti-inflammatory action of IVIg and cytokine blockers on these innate cells [45].

Because of their role in sensing microbes and mediating the inflammatory response, toll-like receptors (TLR), including TLR-4, occupy a central place in the pathogenesis of IBD. Dysregulated TLR signaling due to mutations or abnormal intestinal microbiota is a common feature of IBD, in particular, *E. coli* and *E. faecalis.* During chronic intestinal inflammation, TLR-4 expression is increased and in addition to promoting inflammation, it also stimulates colon carcinogenesis [46]. In the present study, we observed that TLR-4 expression was correlated with increased *E. coli* population while following the IVIg treatment, the expression of TLR-4 was decreased, concurring with the histological and clinical scores data. Altogether, IVIg treatment reduces intestinal inflammation in mice and eliminates *C. albicans* overgrowth from the gut in association with suppression of inflammatory cytokine IL-6 and enhancement of IL-10. Further work is necessary to identify whether protection is associated with alterations in the adaptive immune compartments and the underlying mechanisms, particularly the relative contribution of Fc versus F(ab’)_2_ fragments in mediating the anti-inflammatory activity.

## 4. Methods

### 4.1. Animals

Female C57BL/6 mice (8–10-weeks-old) were purchased from Charles River Laboratories (France). Two complete experimental series were carried out independently. Mice were divided into four control groups, including mice receiving water (CTL), *C. albicans* (Ca), IVIg and DSS (D) alone, and two experimental groups, including *C. albicans* + DSS (DCa) and *C. albicans* + DSS + IVIg (DCaIVIg). All experiments were performed according to protocols approved by the subcommittee for Research Animal Care of the Regional Hospital Centre of Lille, France and the French Ministry of Post-Graduate Education and Research (00550.05, 8/2/2016), and in accordance with European legal and institutional guidelines (86/609/CEE) for the care and use of laboratory animals.

### 4.2. Yeast Strain, Inoculum Preparation, and Induction of Colitis

*C. albicans* SC5314 strain was maintained at 4 °C in yeast peptone dextrose broth (YPD; 1% yeast extract, 2% peptone, 2% dextrose). Each mouse was inoculated on day 1 by oral gavage with 200 µL of PBS containing 5 × 10^7^ live *C. albicans* cells. Mice were given 2% DSS (MW 36−50 kDa; MP Biomedicals, LLC, Germany) in drinking water from day 1 to day 14 to induce intestinal inflammation. For IVIg treatment, mice were administered IVIg Sandoglobulin^®^ (CSL Behring SA; Lot No. 4319800016) intraperitoneally on day 1 (0.8 g/kg) for 7 days. Sandoglobulin^®^ contains at least 96% IgG (typically 99%) with the distribution of the IgG subclasses closely resembling that in normal human plasma. In terms of the subclass distribution, Sandoglobulin^®^ contains 64.5% IgG1, 32.4% IgG2, 2.3% IgG3, and 0.8% IgG4. The product also contains traces of immunoglobulin A (IgA) and immunoglobulin M (IgM).

The presence of *C. albicans* in the intestinal tract was monitored daily by measuring the number of colony-forming units (CFUs) in feces (approximately 0.1 g/sample) collected from each animal. Fecal samples were suspended in 1 mL saline, ground in a glass tissue homogenizer, and plated onto Candi-Select medium (Bio-Rad Laboratories, Marnes la Coquette, France). The CFU of *C. albicans* were counted after 48 h incubation at 37 °C. The results were expressed as CFU/µg of feces. To assess *C. albicans* colonization in the gut, animals were sacrificed, and the GI tract was removed and separated into the stomach, ileum, and colon. The tissues were cut longitudinally. After removal of the intestinal contents, the tissues were washed several times in PBS to minimize surface contamination from organisms present in the lumen. Serial dilutions of homogenates were performed. The results were noted as *C. albicans* CFU/mg of tissue [7,8].

For the isolation of *E. coli* and *E. faecalis* populations, the colonic luminal contents were plated at day 14 onto MacConkey agar (Sigma-Aldrich, St. Quentin Fallavier, France) and Bile esculin azide agar (BEA; Sigma-Aldrich, St. Quentin Fallavier, France). Serial dilutions of these samples were realized. The agar plates were incubated at 37 °C and examined 24 h and 48 h later. Fluconazole (Fresenius Kabi, Louviers, France; 60 mg/L) was added to these two-aerobic media to eliminate the fungal growth cells. For the identification of *E. coli* and *E. faecalis*, a volume of 1.5 μL of matrix solution (α-cyano-4-hydroxycinnamic acid [HCCA]; Bruker Daltonics, Leipzig, Germany) dissolved in 50% acetonitrile, 47.5% water, and 2.5% trifluoroacetic acid was added to each bacterial colony and allowed to dry prior to analysis by MALDI-TOF MS (Microflex-Bruker Daltonics, Bruker Daltonics, Leipzig, Germany).

### 4.3. Assessment of Clinical and Histological Scores

The body weight of each tagged mouse was recorded daily, and the stool consistency and the presence of blood in the rectum were also assessed [47]. Clinical scores, as described previously, were assessed independently by two investigators blinded to the protocol [7,48]. Two scores (stool consistency and bleeding) were added resulting in a total clinical score ranging from 0 (healthy) to 12 (the maximal activity of colitis). Histological scoring was determined by two independent investigators blinded to the protocols. The two subscores (the infiltration of inflammatory cells and the epithelial damage) were added, with the combined histological scores ranging from 0 (no changes) to 6 (extensive cell infiltration and tissue damage) [47,49].

### 4.4. Real-Time mRNA Quantification of Innate Immune Receptors

Total RNA was isolated from the colon using a commercial kit (Nucleospin RNA/Protein; Macherey-Nagel, France). Proteins and mRNA are obtained from the same colon sample and not from two portions of the same sample using this kit. RNA quantification was performed by spectrophotometry (Nanodrop; Nyxor Biotech, France). Reverse transcription of mRNA was carried out in a final volume of 20 µL from 1 µg total RNA (high capacity cDNA RT kit; Applied Biosystems, Villebon Sur Yvette, France). cDNA was amplified by PCR using Fast SYBR green (Applied Biosystems) in the one-step system (Applied Biosystems). SYBR green dye intensity was analyzed using one-step software. All results were normalized to the reference gene, *POLR2A* [50,51].

### 4.5. Quantification of Cytokines

Representative pro-inflammatory (IL-6) and anti-inflammatory (IL-10) cytokine profiles were selected in this study. Cytokine concentrations in the colons were measured using a commercial ELISA kit according to the manufacturer’s instructions (eBioscience, San Diego, CA, USA). Briefly, a volume of 100 μL/well of capture antibodies (anti-mouse IL-10 antibody and anti-mouse IL-6 antibody) in coating buffer (diluted in PBS) was added in NUNC 96 well ELISA plate. The plate was then incubated overnight at 4 °C. After several washings, blocking buffer with 200 μL/well was added to the plate. After different washings, the colon mouse samples were added. The plates were subsequently incubated with biotinylated anti-IL-10 and anti-IL-6 (eBioscience, San Diego, CA, USA), respectively. One hundred microliters per well of Avidin-horseradish peroxidase (HRP) was added to each well, and the absorbance of each well was determined by using a microplate reader at 450 nm. The data are expressed as pg/mL.

### 4.6. Statistical Analysis

All data are expressed as the mean ± standard deviation (SD) of individual experimental groups. Data were analyzed using the Mann-Whitney U test to compare pairs of groups. Differences were considered significant when the *p*-value was as follows: *p* < 0.05; *p* < 0.01; *p* < 0.001.

All statistical analyses were performed with Prism 4.0 from GraphPad and XLSTAT.
